# Scarcity of Hospital Almoners

**Published:** 1924-10

**Authors:** 


### SCARCITY OF HOSPITAL ALMONERS.

Last year eleven students completed their training as hospital almoners and were granted the Certificate of the Institute, the work of which was materially recognised by an anonymous grant of £280, to be disbursed as scholarships for training for almoners' work. The Council consider that not enough women are entering upon this work to fill the vacancies that are likely to occur in the future. Hitherto, indeed, the demand for trained almoners has always exceeded the supply. This is no doubt explained by the fact that the inherent qualities that go to the making of a good almoner are somewhat exceptional. Training will develop these qualities, but it will not create them. A good education, preferably of University standard, is essential.

### Almoner's Department at St. Thomas's.

The Lady Almoner (Miss A. E. Cummins) of St. Thomas's reports that the contributions from and on behalf of patients have steadily increased during the year, although nearly two-thirds are found to be unable to contribute and are urged not to do so when they offer. The enquiry which is now being carried on at this Hospital as to the prevalence and causes of rheumatism and chorea in children demonstrates the need for physician and social worker to combine in their survey of each individual case if the result of the research is to be made as comprehensive as possible. The housing conditions, the character and the sense of responsibility of the parent must be "within the terms of reference" if the physician is to have a full survey of possible causes before compiling his report. This is but one example of the co-operation which is so helpful in modern hospital work, and the wide fields at present untouched call for continued effort to raise the standard of efficiency.

**"Bug Destroyer to the Nobility and Gentry."**

"In 1861 a commercial firm assumed the title of 'Bug Destroyers' and the head of the business announced that he worked for 'the upper classes only, and I have noblemen's names, the first in England, on my books.'"—Sir George Newman.

